# HMMF: a hybrid multi-modal fusion framework for predicting drug side effect frequencies

**DOI:** 10.1186/s12859-024-05806-6

**Published:** 2024-05-20

**Authors:** Wuyong Liu, Jingyu Zhang, Guanyu Qiao, Jilong Bian, Benzhi Dong, Yang Li

**Affiliations:** 1 College of Computer and Control Engineering, Northeast Forestry University, Harbin, 150006 China; 2 Department of Neurology, The Fourth Affiliated Hospital of Harbin Medical University, Harbin, 150001 Heilongjiang China; 3 Computer Science and Technology, Harbin Institute of Technology, Harbin, 150001 China

**Keywords:** Drug repositioning, Drug-side effect frequency, Multi-modal fusion framework, Molecular structures, Biomedical semantics

## Abstract

**Background:**

The identification of drug side effects plays a critical role in drug repositioning and drug screening. While clinical experiments yield accurate and reliable information about drug-related side effects, they are costly and time-consuming. Computational models have emerged as a promising alternative to predict the frequency of drug-side effects. However, earlier research has primarily centered on extracting and utilizing representations of drugs, like molecular structure or interaction graphs, often neglecting the inherent biomedical semantics of drugs and side effects.

**Results:**

To address the previously mentioned issue, we introduce a hybrid multi-modal fusion framework (HMMF) for predicting drug side effect frequencies. Considering the wealth of biological and chemical semantic information related to drugs and side effects, incorporating multi-modal information offers additional, complementary semantics. HMMF utilizes various encoders to understand molecular structures, biomedical textual representations, and attribute similarities of both drugs and side effects. It then models drug-side effect interactions using both coarse and fine-grained fusion strategies, effectively integrating these multi-modal features.

**Conclusions:**

HMMF exhibits the ability to successfully detect previously unrecognized potential side effects, demonstrating superior performance over existing state-of-the-art methods across various evaluation metrics, including root mean squared error and area under receiver operating characteristic curve, and shows remarkable performance in cold-start scenarios.

## Introduction

Adverse drug reactions are a leading cause of drug trial failures during drug development and can have serious consequences on patient health. Severe ADRs (Adverse Drug Reaction) can lead to hospitalizations, long-term medical complications, and even fatalities [1]. Numerous drug side effects are challenging to detect during early development, and some may remain undiscovered for many years even after the drugs have been introduced to the market. Regulators mandate extensive experimentation to assess the safety and effectiveness of drugs before granting approval. Thus, early detection of potential side effects in the drug development cycle is important [2, 3]. However, traditional methods of detecting drug side effects, including clinical trials, double-blind studies, and wet laboratory experiments, are always expensive and time-consuming. In contrast, computational methods [4] provide a quicker and more cost-effective means of uncovering potential side effects [5]. These computational approaches serve two main objectives: predicting side effects for drugs already on the market and identifying potential side effects of new drugs.

In recent years, significant advancements in computational methods have provided researchers with a deeper understanding of the mechanisms behind drug side-effect interactions. This newfound knowledge holds the promise of guiding the development of safer and more effective drugs. Researchers have introduced various computational methods for predicting drug-related side effects [6–9], which can be roughly categorized into two groups: machine learning based and graph representation learning based methods.

Traditional machine learning methods utilize features derived from chemical structures of drugs and biomedical information, employing various classification models for prediction [8, 10]. Additionally, matrix factorization and recommendation algorithms have been extensively used to predict drug-related side effects [11]. Zhang et al. [12] incorporated biomedical information into the matrix factorization framework by applying graph regularization based on drug combination features. Galeano et al. [13] were pioneers in introducing the task of predicting the frequency of drug-related side effects. They proposed a method using non-negative matrix decomposition inspired by recommendation systems, enabling interpretable predictions of potential frequencies. However, their method heavily relies on established frequency relationships and cannot make predictions for a novel drug without any known adverse effects.

In recent years, deep learning models have shown a promising prospect in extracting more complex features of drugs and side effects [14, 15], resulting in improved prediction accuracy compared to traditional machine learning techniques. Dey et al. [16] used a chemical fingerprint algorithm to transform each drug into a 2D or 3D graphical structure, which was compressed into a condensed feature vector through convolution. They employed a fully connected neural network to predict associations between drugs and specific side effects based on the final fingerprint representation for each drug.

In addition to drug features, interactions involving drugs, side effects, and diseases are also crucial. Hu et al. [17] introduced a method for predicting drug-related side effects using a heterogeneous network that integrates various interaction data.They represented the correlations between drugs and side effects as a network graph, synthesizing each node’s representation from its adjacent nodes. Xuan et al. [18] developed heterogeneous graphs based on drug-disease associations and medicinal chemical substructures, unifying specific and common topologies and pairwise attributes of drugs and side effects. However, simplifying identification of drug side effects as a binary prediction task oversimplifies their complexity. Prioritizing side effects with higher frequencies in predictions can streamline drug development in clinical practice. Therefore, there is growing interest in predicting the frequency of drug side effects through regression.

Xu et al. [19] proposed a graph-based attention network approach to learn representations of drugs and side effects based on drug molecular structures and side effect semantics, aiming to predict the frequency of side effects for new drugs with limited available information. On this basis, Wang et al. [20] introduced attribute information, such as drug-gene ontology associations and drug structure associations, and proposed a method for regularizing the frequency of side effects in the neighborhood. Zhao et al. [21] used a graph attention network to integrate three different types of features to extract different view representation vectors: similarity information, known frequency distribution, and word embeddings. These vectors were combined to form a unified prediction vector. To incorporate more information about drugs and side effects, Zhao et al. [22] employed various heterogeneous and homogeneous similarity matrices of drugs and side effects, learning representations through a convolutional neural network channel and two multi-layer perceptron channels.

Zhao et al. [23] provided a detailed summary of recent advances in drug-drug prediction models based on machine learning and deep learning methods, and delved into three score function-based drug-drug prediction models. Meanwhile, Chen et al. [24] comprehensively reviewed drug-target prediction methods based on network and machine learning techniques. Pang et al. [25] and Chen et al. [26] integrated multimodal information to learn deep drug representations. Inspired by these studies, we realize that rich contextual information is embedded in drugs and their associated side effects. Surprisingly, prior studies have not explored the incorporation of textual data, such as drug and side effect descriptions, as new modalities in this context. Especially concerning side effects, the majority of existing studies do not utilize the inherent semantics of the side effects; rather, they simply consider them as category labels for modeling. Furthermore, existing research primarily revolves around binary classification tasks to determine whether drugs are related or not, or regression models to calculate relevant scores, with little exploration of the complementarity between these two tasks.

To address these limitations, we introduce the Hybrid Multi-Modal Fusion (HMMF) framework for predicting drug side effect frequencies. The HMMF model facilitates concurrent multi-modal learning and modeling of the molecular structures, biomedical semantics, attribute similarity features of drugs and side effects. First, we simultaneously conduct context-based representation learning for both drug and side effect description texts. We employ a graph attention network for structural representation learning of drug molecules. Additionally, we investigate similarity learning for drug and side effect attributes. Finally, we utilize a hybrid fusion strategy to merge the five representations derived from these three modalities. Our model benefits from the mutual enhancement between multi-modal and hybrid-fusion strategy. We compared our model with several baseline methods on publicly available datasets and found that our model achieved state-of-the-art experimental results on both tasks. We also conducted ablation experiments to demonstrate the effectiveness of each component of the model.

## Method

### Preliminary

To establish the groundwork for outlining the steps of our method, we first give a clear problem definition and introduce essential notations crucial for predicting the frequency of drug-side effect pairs. Consider a dataset $\mathcal{D}\mathcal{S}$, consisting of triplets (*d*, *s*, *y*), where each triplet denotes a drug, its associated side effect, and the frequency of occurrence, i.e., $\mathcal{D}\mathcal{S} = {(d, s, y)_i}$. $D = {d_1, d_2, \ldots , d_n}$ represents the set of drugs, and $S = {s_1, s_2, \ldots , s_m}$ is the set of side effects. To predict the frequency of drug-related side effects, a regression model is employed to approximate the actual frequency closely. If drug $d_i$ and side effect $s_j$ in matrix $A \in \mathbb {R}^{n \times m}$ exhibit correlation, the resulting *y*-value is assigned one of five scores, ranging from 1 to 5. These scores are categorized as ${\textbf {very rare}}$ (frequency = 1), ${\textbf {rare}}$ (frequency = 2), ${\textbf {infrequent}}$ (frequency = 3), ${\textbf {frequent}}$ (frequency = 4), and ${\textbf {very frequent}}$ (frequency = 5). In cases where $d_i$ and $s_j$ are unrelated, $A(i, j) = 0$.

**Fig. 1:**
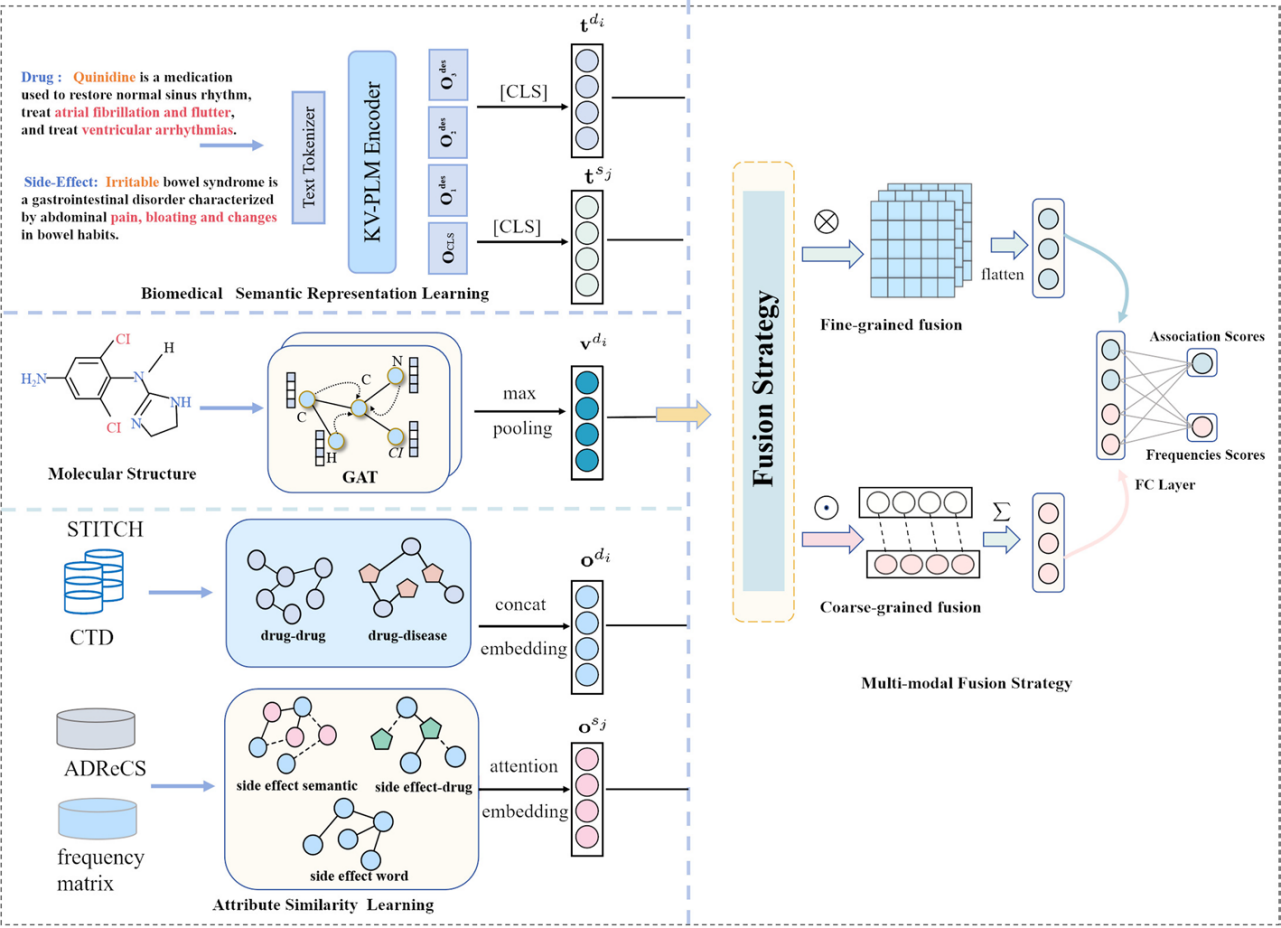
The proposed hybrid multi-modal fusion (HMMF) framework for predicting drug side effect frequencies

Next, we will provide a detailed description of our approach to predict the frequency of drug side effects. As shown in Fig. 1, our method comprises four components: Biomedical Semantic Representation Learning, Molecular Structure Representation Learning, and Attribute Similarity Learning, and Multi-modal Fusion Strategy.

### Biomedical semantic representation learning

**Fig. 2:**
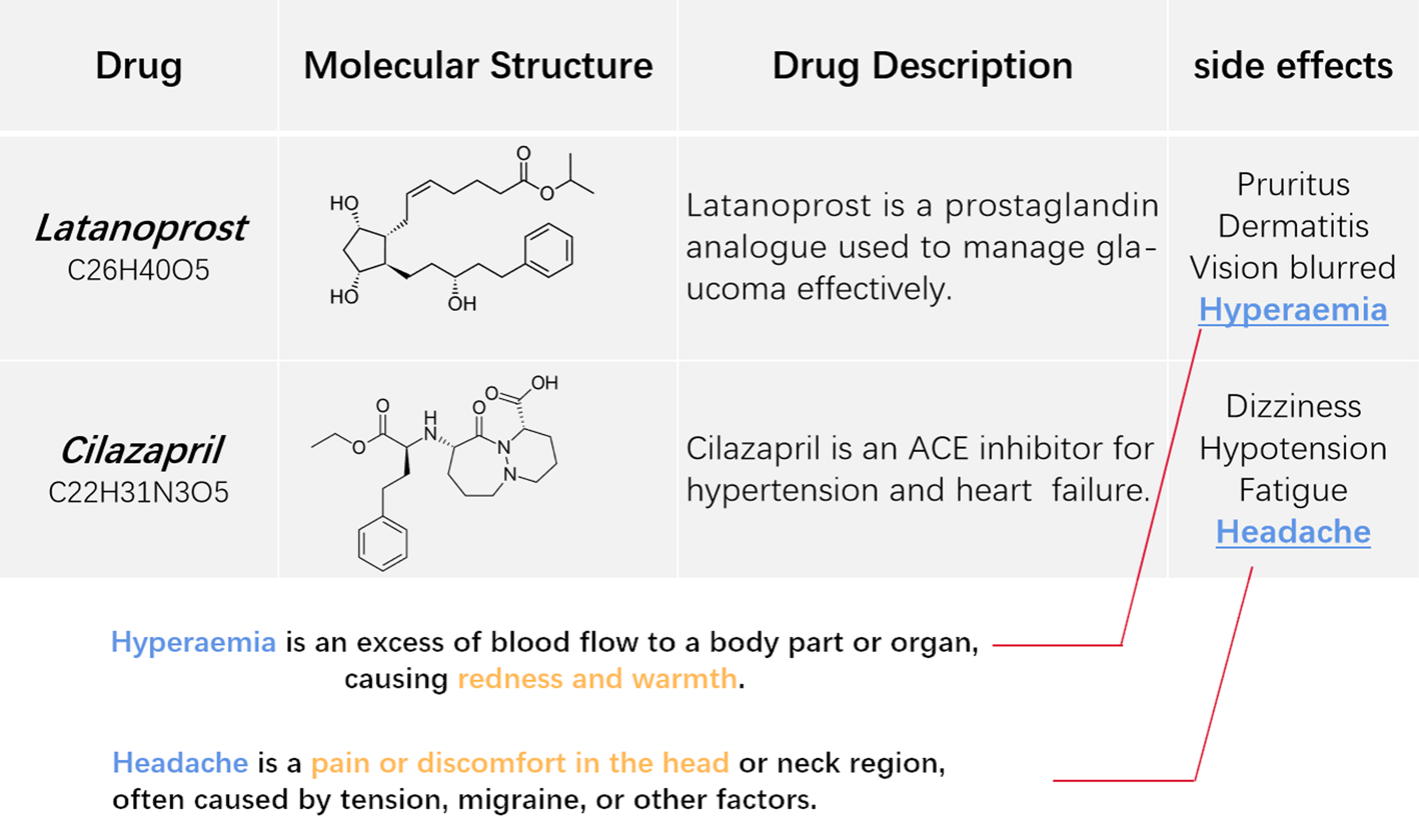
Examples describe the drugs latanoprost and cilazapril, and the side effects of hyperaemia and headache collected from different data sets (such as Wikipedia and Punched)

We collect biomedical text information for drugs and side effects from Wikipedia and PubChem, as shown in Fig. 2. To prevent potential data leakage, all descriptions involving interactions between drugs and side effects were excluded from the collected biomedical texts. For example, sentences like *“Etoposide often causes nauea, vomiting, and loss of appointment”* were not included in the biomedical text data.

Let $p^{d_{i}}=\{w^{d_{i}}_{1},w^{d_{i}}_{2},w^{d_{i}}_{3},\ldots ,w^{d_{i}}_{n}\}$ represent the biomedical text information of drug $d_i$, $k^{s_{j}}=\{w^{s_{j}}_{1},w^{s_{j}}_{2},w^{s_{j}}_{3},\ldots ,w^{s_{i}}_{n}\}$ represent the biomedical text information of side effect $s_j$. We employ a multi-modal pre-training language model, ${\text {KV-PLM}}$ [27], to learn the contextual representation of biomedical text information for drugs and side effects. We selected ${\text {KV-PLM}}$ because it concurrently learns molecular structures and biomedical texts during pre-training, facilitating the integration of multiple information sources and enhancing the extraction of more comprehensive features for drugs and side effects. Subsequently, we extract the embedding of the entire sentence, denoted as $\textbf{O}_{cls}$, to represent the semantic information of drugs and side effects. The biomedical semantic representation of drug $d_i$ and side effect $s_j$ can be obtained as follows:

1 $$\begin{aligned} \textbf{t}^{d_{i}}={\text {KV}}{} {\textbf {-}}{\text {PLM}} \left( p^{d_{i}} \right) ,\textbf{t}^{s_{j}}={\text {KV}}{} {\textbf {-}}{\text {PLM}} \left( k^{s_{j}} \right) , \ \ \ \textbf{t}^{d_{i}},\textbf{t}^{s_{j}} \in \mathbb {R}^{N \times f} \end{aligned}$$

where *N* is The number of drugs or side effects, *f* is the output dimension of ${\text {KV-PLM}}$.

### Molecular structure representation learning

Previous studies [28] have highlighted the effectiveness of the graph attention network (GAT) in extracting representation for drug molecular structures. GAT employs an attention mechanism to more accurately evaluate the contributions of neighboring nodes to the target node, enabling a more comprehensive consideration of the global information within the molecular graph. Building upon this prior work, for drug $d_{i}$, we use the RDKit tool to convert the SMILES (Simplifed Molecular Input Line Entry System) sequence into an undirected molecule graph $G_{i} = (V, E)$. Here, $V = \{C, H, O, \ldots , Sr\}$ represents the atomic types, and *E* represents the set of chemical bonds between the atoms. Each atom in the compound for drug $d_{i}$ possesses an attribute vector $X_i \in \mathbb {R}^{m \times 1}$, initialized based on the attribute values corresponding to each dimension. Subsequently, we build the molecular topology graph $\mathcal {G}_{i} = (\textbf{A}_{i}, \textbf{X}_{i})$, where $\textbf{A}_{i} \in \mathbb {R}^{n \times n}$ represents the adjacency matrix of $\mathcal {G}_{i}$, and $\textbf{X}_{i} \in \mathbb {R}^{n \times m}$ is the matrix containing atomic features. In this context, *n* denotes the number of atoms in drug $d_i$, while *m* is the dimension of the feature vector for each atom.

The similarity between the target atom node *p* and its neighbor atom node *q* ($q \in \mathcal {N}_p$) can be calculated as follows:

2 $$\begin{aligned} \textbf{e}_{pq}=\textbf{H}\left( \textbf{W} \textbf{X}_p ; \textbf{W} \textbf{X}_q\right) , q \in \mathcal {N}_p \end{aligned}$$

where $\textbf{W}$ represents a learnable parameter matrix, while $\textbf{H}$ $\in \mathbb {R}^{2d}$ denotes the dimensions of the hidden layers in GAT. $\textbf{X}_{(.)}$ is the one-hot vector of the atomic node. $\mathcal {N}_p$ stands for the set of neighboring nodes of node *p*, and ;  represents the concatenation operation.

Next, we utilize the softmax function to normalize all neighboring nodes of atom node *p*, which can be expressed as follows:

3 $$\begin{aligned} \alpha _{p q}= & {} {softmax}\left( \textbf{e}_{p q}\right) =\frac{\exp \left( {\text {LeakyReLU}}\left( \textbf{e}_{p q}\right) \right) }{\sum _{k \in \mathcal {N}_p} \exp \left( {\text {LeakyReLU}}\left( \textbf{e}_{p k}\right) \right) }\end{aligned}$$

4 $$\begin{aligned} \mathbf {v_p}= & {} \Vert _{h=1, \ldots , l}\ \sigma \left( \sum _{q \in \mathcal {N}_p} \alpha _{pq}^{(h)} \textbf{W} \textbf{X}_q\right) \end{aligned}$$

where $||{h=1, \ldots , l}$ denotes the output of multiple attention heads, and (*l*) signifies the total number of attention heads that we have defined. ${\Vert} _{h=1, \ldots , l}$is the concatenation of the outputs from different heads. Lastly, the drug molecular structure representation $\textbf{v}^{d_{i}}$ of drug $d_i$ is obtained by applying max pooling to the embedding of each atom.

### Attribute similarity learning

In addition to extracting embeddings from the rich structural and bio-semantic information of drugs and side effects, we can also learn existing attribute similarity information to capture the profound relationship between drugs and side effects.

#### Drug similarity

We collect drug-related data from two primary sources: the STITCH database, which provides drug chemistry structures, and the Comparative Toxicogenomics Database (CTD), which details drug-disease associations.

The STITCH database is a comprehensive resource for exploring drug-chemical interactions, providing detailed information on the chemical structures of various drugs. It primarily constructs an association matrix, $\textbf{S}_{\text {drug-chem}} \in \mathbb {R}^{N{\text {drug}}\times N_{\text {drug}}}$, that captures similarity scores among drug compounds. This matrix, with dimensions, provides valuable insights into the chemical resemblances among different drugs within our dataset. Conversely, the CTD database serves as a vital repository of associations between drugs and diseases. The CTD database collects extensive data, capturing 330,397 associations across 750 drugs and 6,808 diseases from benchmark datasets. These associations are meticulously represented in a drug-disease association matrix, denoted as $\mathbf {S'}_{\text {drug-disease}}$, where each entry *s*(*i*, *j*) signifies the relationship between drug *i* and disease *j*, with *s*(*i*, *j*) serving as a binary indicator (0 or 1) of association presence. Subsequently, we calculate the Jaccard similarity between the rows and columns of $\mathbf {S'}_{\text {drug-disease}}$, facilitating the construction of a similarity matrix denoted as $\textbf{S}_{\text {drug-disease}} \in \mathbb {R}^{N_{\text {drug}}\times N_{\text {drug}}}$.

After obtaining the two attribute similarity matrices for drugs, to derive the representation of a drug, we can concatenate the *i*-th row of $\textbf{S}_{\text {drug-chem}}$ and $\textbf{S}_{\text {drug-disease}}$ as the initial feature representation for drug $d_i$. Subsequently, we project the representation into the same space as that of side effects, the drug similarity representation of $d_i$ is denoted as $\textbf{o}^{d_i} \in \mathbb {R}^{1 \times dim}$.

5 $$\begin{aligned} \textbf{o}^{d_i}=\sigma \left( \textbf{W}\left( \textbf{S}_{\text{ drug-chem } }[i,:] ; \textbf{S}_{\text{ drug-disease } }[i,:]\right) +b\right) \end{aligned}$$

where [*i*,  : ] represents the i-th row of the matrix, and ;  denotes concatenation operation.

#### Side effect similarity

To measure the similarity of hyponymy among side effects, we retrieve the relevant data from the ADReCS database to initialize our side effects [29]. This database is organized with a four-level tree structure, where each ADR item is given a unique ID. For example, in the ADReCS dataset, polycythemia is identified with the unique ID $\textit{14.12.01.002}$. We have constructed a directed acyclic graph (DAG), with nodes representing side effects and links denoting relationships [30]. In this graph, the only type of relationship is defined as ‘is-a’, connecting child nodes to parent nodes. We define the contribution of a side effect *s* in ${\textbf {DAG}}_A$ to the semantics of side effect *A* as the *D* value associated with side effect *s* concerning side effect *A*.

6 $$\begin{aligned} D_A(s)=\left\{ \begin{array}{cl} 1 &{} \text{ if } s=A \\ \max \left\{ \mu *D_A\left( s^{\prime }\right) \mid s^{\prime } \in {\text {children}}(s)\right. &{} \text{ if } s \ne A\} \end{array}\right. \end{aligned}$$

where $\mu$ represents a fixed weight for the semantic contribution value. We have set $\mu$ to 0.5 based on the practical experience outlined in the previous work. Consequently, we can compute the total semantic value of side effect *A* using the following formula:

7 $$\begin{aligned} {DV}(\textrm{A})=\sum _{t \in {Anc}(\textrm{A})} D_{\textrm{A}}(t) \end{aligned}$$

where *Anc*(*A*) refers to a set of nodes comprising all ancestor nodes of side-effect *A*, including *A* itself. Typically, the closer an ancestor node is to *A*, the greater its contribution will have on *A*, and vice versa.

Then, for a pair of side effect $s_i$ and $s_j$, the similarity of hyponymy among them can be defined as follows:

8 $$\begin{aligned} {\textbf{sim}}\left( s_i, s_j\right) =\frac{\sum _{x \in {Anc}\left( s_i\right) \cap {Anc}\left( s_j\right) }\left( D_{s_i}(x)+D_{s_j}(x)\right) }{DV\left( s_i\right) +DV\left( s_j\right) } \end{aligned}$$

Finally, we construct the hyponymy similarity matrix of side effects, denoted as $\textbf{S}_{\text {side-hypo}} \in \mathbb {R}^{N_{\text {side effect}}\times N_{\text {side effect}}}$.

Using a pre-trained word2vec model based on Wikipedia, embeddings are generated for each side effect term in the benchmark dataset, constructing a side effect feature matrix $\mathbf {S'}_{\text {side-word}} \in \mathbb {R}^{N_{\text {side effect}}\times \text {f}}$, where *f* is the output dimensionality of the word2vec model. Subsequently, by computing the cosine similarity between side effects, these representations are utilized to build a matrix of word similarities for side effects, represented as $\textbf{S}_{\text {side-word}} \in \mathbb {R}^{N_{\text {side effect}} \times N_{\text {side effect}}}$.

To make full use of the known drug-side effect association information, we transpose the drug-side effect association matrix and, based on the transposed matrix, calculate cosine similarity to construct a similarity matrix for side effects $\textbf{S}_{\text {side-drug}} \in \mathbb {R}^{N_{\text {side effect}}\times N_{\text {side effect}}}$.

We extract the *j*-row in the similarity matrices $\textbf{S}_{\text {side-hypo}}$, $\textbf{S}_{\text {side-word}}$ and $\textbf{S}_{\text {side-drug}}$. We assign different weights to these rows for constructing the initial feature representation of the side effect $s_j$. The specific weight formula is as follows:

9 $$\begin{aligned} \alpha ^{_{\text {side-hypo}}}={\text {softmax}}\left( \tanh \left( \textbf{W} \cdot \left( \textbf{u}^{_{\text {side-hypo}} }\right) ^{\intercal }+b\right) \right) \end{aligned}$$

where $\alpha ^{_{\text {side-hypo}}}$ is the weight of $\textbf{u}^{_{\text {side-hypo}}}$, $\textbf{W}$ and *b* are learnable parameters. Similarly, we can obtain $\alpha ^{_{\text {side-word}}}$ and $\alpha ^{_{\text {side-drug}}}$. Finally, the representation of side effect similarity representation is:

10 $$\begin{aligned} \textbf{o}^{s_j} = \alpha ^{_{\text {side-hypo}} }\cdot {\textbf{u}^{_{\text {side-hypo}}}} + \alpha ^{_{\text {side-word}}} \cdot {\textbf{u}^{_{\text {side-word}} }} +\alpha ^{_{\text {side-drug}}} \cdot {\textbf{u}^{_{\text {side-drug}} }} \end{aligned}$$

### Multi-modal fusion strategy

Before integrating different modal representations, we begin by projecting the representations derived from the biomedical semantic modality and the molecular structure modality into a unified space that aligns with the attribute similarity modality. For drug $d_i$, we the biomedical semantic representation $\textbf{t}^{d_i}$, molecular structure representation $\textbf{v}^{d_i}$ and attribute similarity representation $\textbf{o}^{d_i}$. For side effect $s_j$, we have the biomedical semantic representation $\textbf{t}^{s_j}$ and the attribute similarity representation $\textbf{o}^{s_j}$. This unified space is of dimension *dim*.

To facilitate information interaction across different modalities, we design two fusion mechanisms.

**Fusion Strategy 1 (coarse-grained fusion):** Given each representation of drug $\textbf{a}^{d_i} \in \{ \textbf{t}^{d_i}$, $\textbf{v}^{d_i}$, $\textbf{o}^{d_i} \}$, we first perform element-wise product operation with each side effect representation $\textbf{b}^{s_j} \in \{ \textbf{t}^{s_j}, \textbf{o}^{s_j} \}$:

11 $$\begin{aligned} \textbf{c}^{di,sj}_1=\sigma \left( \left( \sum \left( \textbf{a}^{d_i} \bigodot \textbf{b}^{s_j} \right) \right) \textbf{W}\right) \end{aligned}$$

where $\textbf{c}^{di,sj}_1$ represents the learned coarse-grained fusion representation of each drug-side effect pair.

**Fusion Strategy 2 (fine-grained fusion):** Given each representation of drug $\textbf{a}^{d_i} \in \{ \textbf{t}^{d_i}$, $\textbf{v}^{d_i}$, $\textbf{o}^{d_i} \}$, we perform the outer product operation with each side effect representation $\textbf{b}^{s_j} \in \{ \textbf{t}^{s_j}, \textbf{o}^{s_j} \}$:

12 $$\begin{aligned} \textbf{c}^{di,sj}_2=\sigma \left( \left( \textrm{CNN} \left( \textbf{a}^{d_i} \times \textbf{b}^{s_j} \right) \right) \textbf{W}\right) \end{aligned}$$

where $\textrm{CNN}$ (Convolutional Neural Network) is an encoder commonly used in image representation learning to extract fine-grained features. We utilize it in our approach to learn fine-grained fusion representation of each drug-side effect pair.

### Loss function

Up to this point, we have acquired both the coarse-grained and fine-grained fusion representations of the drug-side effect pair, denoted as $\textbf{c}^{di,sj}_1$ and $\textbf{c}^{di,sj}_2$. We concatenate these two representations and input them into a two-layer fully connected neural network to generate the predicted frequency score and association score for drug side effects in this model.

13 $$\begin{aligned} FS^{d_i,s_j}={\text {MLP}}\left( {\textbf{c}^{d_i,s_j}_1};{\textbf{c}^{d_i,s_j}_2}\right) \end{aligned}$$

where $FS^{d_i,s_j}$ is the frequency score of drug $d_i$ and side effect $s_j$.

14 $$\begin{aligned} AS^{d_i, s_j}={\text {Sigmoid}}\left( {\text {MLP}}\left( \textbf{c}^{d_i,s_j}_1 ; \textbf{c}^{d_i,s_j}_2\right) \right) \end{aligned}$$

where $AS^{d_i, s_j}$ is the association score between drug $d_i$ and side effect $s_j$.

Our proposed method, illustrated in Fig. 1, yields two scores: the probability of association between drug-side effect pairs and the frequency score when making predictions for positive samples. The objective function of HMMF is as follows:

15 $$\begin{aligned} \mathcal {L}_{1}= & {} \sum _{i=1}^n\left( AS^{d_i, s_j}- \hat{k} \right) ^2,\mathcal {L}_{2}=\sum _{i=1}^n\left( FS^{d_i, s_j}-\hat{y}\right) ^2 \end{aligned}$$

16 $$\begin{aligned} \mathcal {L}= & {} \mathcal {L}_{1}\times \mathcal {L}_{2}+\gamma R(\Theta ) \end{aligned}$$

where $\hat{k} \in (0,1)$ represents the ground-truth association score of the drug side effect pair, and $\hat{y} \in \{1,2,3,4,5\}$ represents the ground-truth frequency score. $R(\Theta )$ corresponds to the L2 regularization term, which is the sum of the squared weight values, where $\Theta$ encompasses all trainable model parameters. Additionally, $\mathcal {L}{1}$ and $\mathcal {L}{2}$ are loss functions designed to minimize the association and frequency errors between drugs and side effects.

## Results

In this section, we explore the feasibility and effectiveness of the proposed model in predicting the frequency of drug side effects through experiments. Specifically, we address the following research questions: RQ1. Is the proposed multimodal fusion model both feasible and effective? RQ2. If so, which modules contribute more significantly to its enhancement? RQ3. How does the model perform when encountering data on new drugs?

### Dataset

The frequency information of drug side effects in the benchmark dataset is obtained from the SIDER database and collected by Galeano [13]. The dataset contains 37,071 known frequency pairs of drug side effects, covering 750 drugs and 994 side effects. There are five frequency scores for drug side effects, including very rare (frequency = 1), rare (frequency = 2), uncommon (frequency = 3), frequent (frequency = 4), and very frequent (frequency = 5). We have observed that the majority of known frequency pairs of drug side effects are either uncommon or frequent, making the dataset significantly imbalanced.

Additionally, in our proposed model, we introduce association and similarity matrices for various drug and side effect attributes. The drug-disease association data is obtained from the Comparative Toxicology Genome Database (**CTD**), while the similarity score between drugs $d_i$ and drug $d_j$ is sourced from the **STITCH** database. For each drug or side effect, we gather their SMILES sequences and biomedical text information from **Pubchem** and **WIKI**. To obtain side effect information, we utilize the Adverse Drug Reaction Classification System (**ADReCS**).

### Baselines

In the comparison experiment, we used the following models as baselines for predicting drug-side effect frequencies. We evaluated the performance of all baseline methods using the same dataset and employed the parameter settings as specified in their respective work.

- **Galeano’s model**[13] introduced a recommendation system-based approach for predicting the frequencies of drug side effects using matrix decomposition methods. Nevertheless, this method has limitations when it comes to forecasting the frequencies of associated side effects for novel or unidentified drugs.
- **MGPred**[21] extracted initial features of drugs and side effects from various heterogeneous datasets. It predicted the frequency of drug side effects by integrating representations from multiple perspectives using an attention network.
- **DSGAT**[19] employed a graph attention network to acquire embeddings for drug molecular graphs and side effect graphs. These two embeddings were mapped into a shared vector space, and matrix decomposition was utilized for decoding. It is worth mentioning that this approach primarily focuses on extracting features from drug molecular structures, which might result in the oversight of other essential features.
- **SDPred**[22] integrated data from diverse sources concerning drugs and side effects to learn embeddings of drug-side effect pairs through multiple channels. The predicted outcomes are generated by inputting these embeddings into a multilayer perceptron.
- **NRFSE** [20] uses class-weighted non-negative matrix factorization to decompose the drug-side effect frequency matrix, employing Gaussian likelihood for modeling unknown drug-side effect pairs. Additionally, it integrates a multiview neighborhood regularization strategy, merging three drug attributes and two side effect attributes to ensure similarity in latent features among the most similar drugs and side effects.

### Experimental setup

In this study, we evaluate the effectiveness of our proposed model and baseline methods using a nested 5-fold cross-validation approach on a standardized benchmark dataset. Positive samples consist of the frequencies of all known drug side effects, with an equal number of unrelated drug side effects randomly selected as negative samples. The combined pool of positive and negative instances is subsequently randomly partitioned into five distinct subsets. During each iteration of the outer validation loop, one subset is designated as the test set, while the remaining four subsets collectively constitute the training set. Within each outer fold, an inner loop employs a five-fold cross-validation procedure to fine-tune model hyperparameters and evaluate performance. Performance metrics reported reflect the average outcomes derived from the nested 5-fold cross-validation procedure.

During the training of our proposed model on an NVIDIA A100 with 80 GB VRAM, we conduct hyperparameter optimization via inner cross-validation. The model’s training epochs are capped at 400. Preliminary experiments are conducted on combinations of learning rate, batch size, and embedding dimensions to observe performance trends. Based on these preliminary results, we select values that demonstrate stability and potential under 5-fold cross-validation: an initial learning rate of 5e-4 with a learning rate decay strategy reducing the rate by 80% after 250 epochs, a batch size of 128, and an embedding dimension of 128. Subsequently, through grid search during inner cross-validation, dropout rates within the range [0.4, 0.5, 0.6] and $\gamma$ values within [1e-3, 1e-4, 1e-5] are explored to determine the optimal hyperparameter combinations for each fold. We specify weight decay as 1e-3. Finally, for the multi-layer convolutional neural network, filter sizes of 2$\times$2 with a stride of 2 are utilized.

### Evaluation metrics

To comprehensively evaluate the performance of various methods, we consider multiple evaluation metrics. Specifically, we use AUPR (Area Under the Precision-Recall curve) and AUROC to evaluate the drug-side effect association performance. We employ RMSE and MAE (Mean Absolute Error) to evaluate drug-side effect frequency prediction performance, where smaller errors indicate better model performance, indicating that the model’s predictions are close to the actual values.

**AUROC**: The AUROC curve is a widely used method for evaluating the performance of binary classification models. It plots the True Positive Rate (TPR) against the False Positive Rate (FPR) at various decision thresholds, demonstrating how well the model distinguishes between positive and negative samples. A larger area under the curve (AUC) is desirable as it indicates predictions with higher accuracy.

**AUPR**: The AUPR stands for the area under the Precision-Recall curve, where the *x*-axis represents the recall rate, and the *y*-axis represents accuracy. In real-world data, the distribution of positive and negative samples is often highly imbalanced, making AUPR a more suitable evaluation metric for evaluating model performance.

**MAE** and **RMSE**: To evaluate the performance of drug-side effect frequency prediction in the regression-based task, we employ evaluation metrics such as root mean square error (RMSE) and mean absolute error (MAE). These statistical measures quantify the error between the actual and predicted values of the samples and are frequently utilized in regression tasks.

17 $$\begin{aligned} \text{ RMSE } =\sqrt{\frac{1}{n}\sum _{i=1}^n\left( {y}_i-{z}_i\right) ^2}, \text{ MAE } =\frac{1}{n} \sum _{i=1}^n|y_i-{z}_i| \end{aligned}$$

where *n* represents the total number of drug-side effect pairs with frequency scores, $y_{i}$ represents the predicted frequency score, and ${z}_{i}$ denotes the ground-truth frequency score.

### Experimental results

**Fig. 3:**
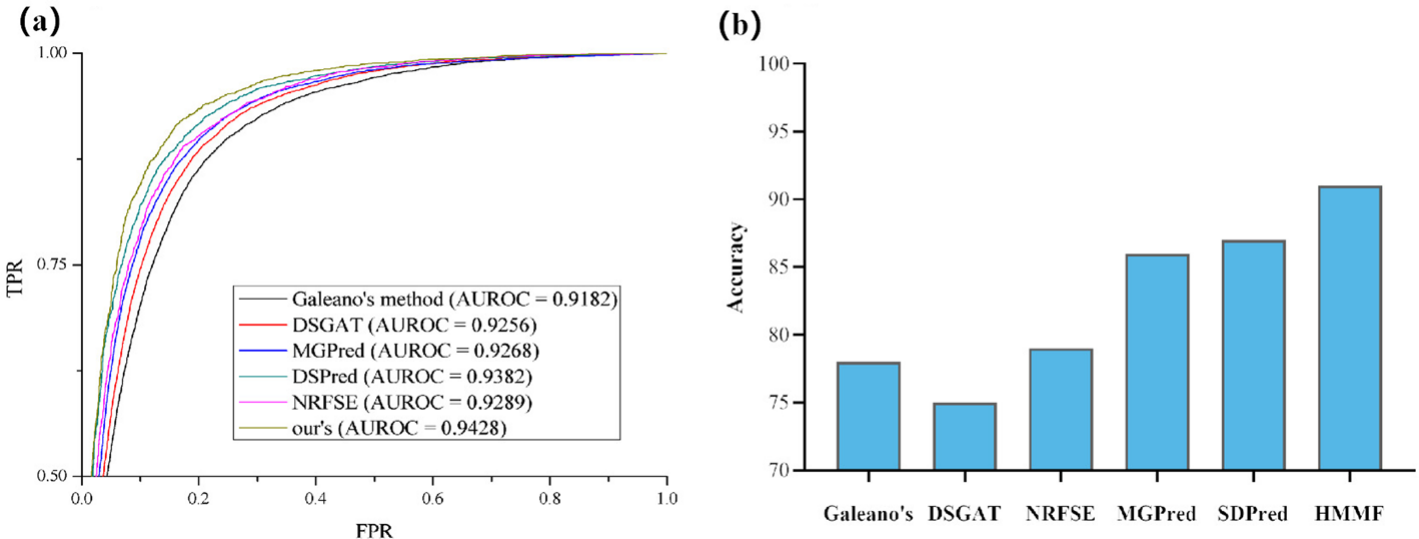
**a** The AUROC curves for all baseline methods and our HMMF model. **b** Accuracy Results of Drug Side Effects Based on High-Scoring Predictions

In Table 1, we compare the experimental results of all baseline methods and our proposed HMMF model. Based on the table, we observe that the HMMF model outperforms all the baseline methods across various performance metrics. We can draw the following conclusions from the results in Table 1 and Fig. 3a: (i) For the AUROC and AUPR metrics, the HMMF model shows a relatively small but excellent performance improvement. Compared to the best-performing baseline method, SDPred, the HMMF model demonstrates an improvement of approximately 0.5% in both metrics. This signifies that the HMMF model achieves higher accuracy and superior classification performance. While our improvements may not be as substantial when compared to SDPred, it is worth noting that SDPred already makes use of a substantial amount of similarity data, providing rich initial association features. (ii) For the RMSE and MAE metrics, the HMMF model’s performance is significantly better than other baseline methods. Notably, the RMSE is reduced by about 1–1.5%, and the MAE is reduced by about 1.5– 2%. These results indicate that the HMMF model excels in predicting errors and estimating accuracy. (iii) Compared to DSGAT, which relies solely on the molecular structures of drugs for learning drug embeddings, our model combines various data sources, such as biomedical texts and multiple attribute similarities between drugs and side effects. This results in significant improvements in both AUROC and AUPR, along with a considerable reduction in RMSE and MAE. These enhancements demonstrate the effectiveness of our approach in capturing drug and side effect relationships and accurately predicting their frequencies.

**Table 1:** The experimental results of all baseline methods and our HMMF model on the benchmark dataset are presented

Model	AUROC	AUPR	RMSE	MAE
Galeano’s method	0.9182	0.9178	1.2980	0.9530
DSGAT	0.9256	0.1893	1.0599	0.7642
MGPred	0.9268	0.9175	0.6635	0.5058
SDPred	0.9382	0.9352	0.6089	0.4375
NRFSE	0.9289	0.1948	0.9882	0.7342
HMMF	0.9428	0.9398	0.5810	0.4216

Higher values indicate better results for AUROC and AUPR, while for RMSE and MAE, lower values are preferred

**Fig. 4:**
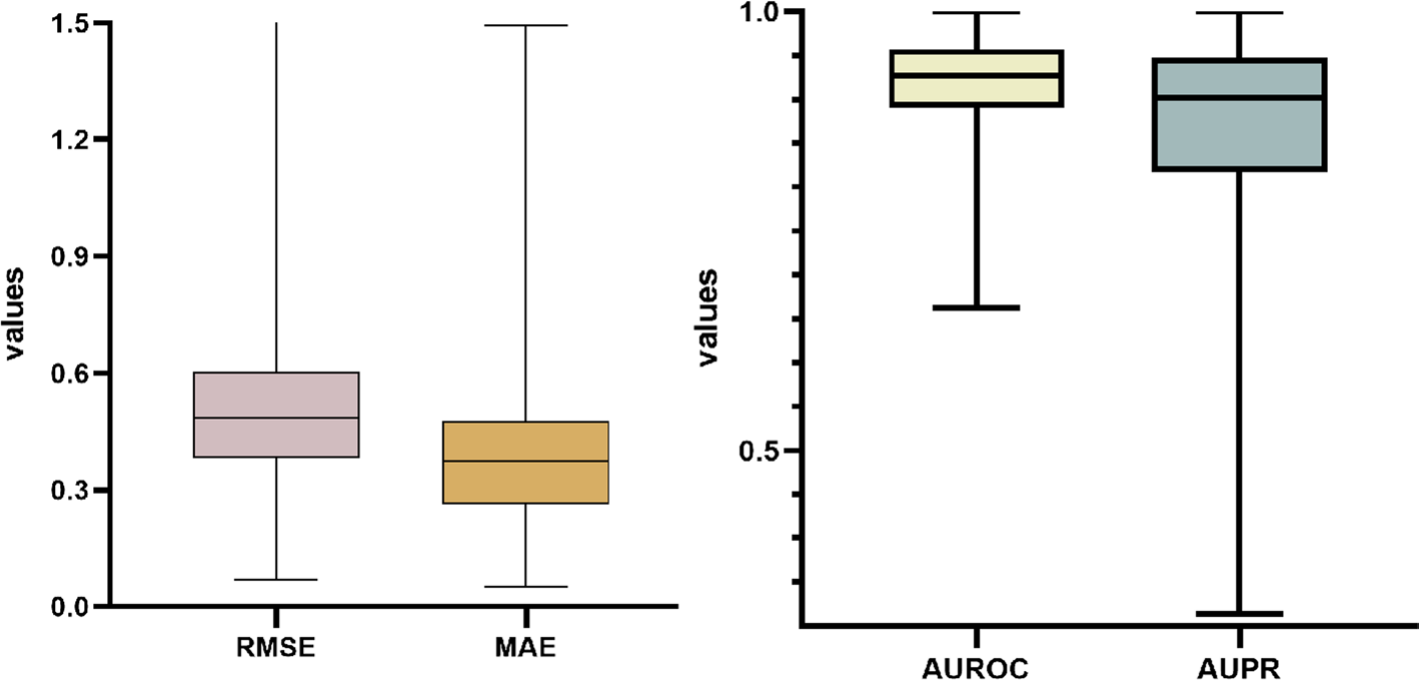
Distribution of RMSE, MAE, AUROC, AUPR values for all drugs in the main experiment

In summary, the HMMF model excels in various metrics, with a particularly notable improvement in RMSE and MAE. These findings demonstrate that the HMMF model provides better predictive performance than other baseline methods, especially in the task of drug-side effect frequency prediction. To further investigate the model’s ability to predict the frequency of side effects for individual drugs, we present distribution of the four evaluation metrics for every in Fig. 4. The average values for AUROC and MAE for all drugs are 0.915 and 0.369, respectively.

To assess the significant advantage of our model over the current state-of-the-art (SOTA) model SDPred, we conducted a two-sided Wilcoxon rank-sum test on all drugs in the benchmark dataset. The results were indeed impressive. Our model achieved significantly lower p-values of $3.547 \times 10^{-07}$ based on AUROC and $2.694 \times 10^{-19}$ based on MAE compared to SDPred, indicating that our model outperforms SDPred with statistical significance. Demonstrating marked improvements in both prediction accuracy and performance, these p-values are well below the conventional significance threshold of 0.05, providing strong statistical evidence of our model’s superiority over SDPred.

### Ablation study

Next, we verify the impact of different model modules by removing them from the full model. “*only* structural formula” indicates that model learning only predicts the frequency of side effects based on the molecular structure of drugs. “*only* biomedical semantic” denotes using solely biomedical texts related to drugs and their associated side effects as input, excluding additional attributes. “ *w*/*o* molecular structure semantic” indicates the model’s performance without considering molecular structure. “ *w*/*o* drug similarity” and “ *w*/*o* side effect similarity” represent the exclusion of attribute similarity for drugs and side effects, respectively. “ *w*/*o* fine-grained fusion” and “ *w*/*o* coarse-grained fusion ” denote the exclusion of different fusion strategies. Table 2 presents the RMSE and MAE results of each module ablation experiment on the benchmark dataset.

We can draw the following conclusions: (*i*) Exclusively incorporating either biomedical text or structural formula input, while excluding other modules in the model, also yielded impressive AUC and AUPR scores. This finding validates our hypothesis regarding the effectiveness of capturing the relationship between drug side effects solely from biomedical text input. It is worth noting that structural characterization shows superior performance in predicting the frequency of side effects compared with drugs with biomedical semantic. (*ii*) Removing information modules such as molecular structure and attribute similarity leads to a decline in overall performance, highlighting the importance of multi-modal fusion in predicting drug side effects. (*iii*) Our approach is distinct in that it employs two fusion mechanisms to integrate drugs and side effects before input, as opposed to directly connecting them to a multilayer perception. This fusion methodology allows for a more effective capture of the intricate relationship between these elements. In summary, the experimental results demonstrate that each module in our proposed model complements the others, ultimately improving the prediction performance of drug side effect frequency.

**Table 2:** Experimental results of our model and its degenerated models

Model	AUROC	AUPR	RMSE	MAE
*only* structural formula	0.8921	0.8968	0.6648	0.4973
*only* biomedical semantic	0.8869	0.8832	0.6790	0.5103
*w*/*o* molecular structure	0.9412	0.9389	0.5927	0.4298
*w*/*o* drug similarity	0.9283	0.9257	0.6012	0.4487
*w*/*o* side-effect similarity	0.9118	0.9098	0.6394	0.4896
*w*/*o* coarse-grained fusion	0.9365	0.9321	0.6074	0.4431
*w*/*o* fine-grained fusion	0.9379	0.9360	0.5920	0.4309
Full HMMF	0.9428	0.9398	0.5810	0.4216

### Cold start analysis

The preliminary assessment of new drugs for predicting adverse effects is a critical concern, especially in the context of clinical trials. New drugs often lack established data on the frequency of adverse effects, making methods like Galeano’s unsuitable for the common cold-start scenarios found in drug discovery. To evaluate the efficacy of our approach in forecasting the incidence rates of adverse effects for new pharmaceuticals within a cold-start setting, we employed the 10-fold cross-validation technique. This method uses a single loop to conduct the cross-validation. During each iteration, models are trained on a subset of the data and then tested on the remaining data.

To ensure fairness in our cold-start experiments, our competitors, MGPred, NRFSE, and SDPred, did not use embeddings derived from drug and side effect association matrices during each fold. Similarly, our model excluded the $\textbf{S}_{\text {side-drug}}$ module, which also derives embeddings through association matrices. We then randomly selected 10% of the drugs from our initial dataset of 750 for the final test phase, while the remaining 90% were used for training within the cross-validation. Notably, in cold-start scenarios, the way data is partitioned significantly affects performance evaluation. Therefore, we maintained consistent data partitioning for the 10-fold cross-validation across all models. The results, as presented in Table 3, demonstrate that our model performs exceptionally well in cold-start scenarios, showing a significant improvement compared to typical conditions. This highlights our model’s robustness and its ability to generalize effectively to unknown drugs.

**Table 3:** Experimental results in cold start drugs

Model Name	AUROC	AUPR	RMSE	MAE
DSGAT	0.8281	0.2915	1.4646	1.1732
MGPred	0.7768	0.2765	0.8960	0.6680
NRFSE	0.8322	0.3265	1.4126	1.1420
SDPred	0.8426	0.3309	0.8549	0.6243
HMMF	0.8679	0.3668	0.7896	0.5548

### Predicting high-frequency drug side effects

To further evaluate the performance of our proposed method, we conducted an additional experiment specifically focusing on the top 100 high-score predictions. The primary aim of this experiment was to assess the accuracy proportion within this dataset and juxtapose the results with other methods. The outcomes of this experiment are depicted in Fig. 3b. Methods such as Galeano’s method, DSGAT, and NRFSE solely predict frequency scores without directly predicting specific associations between drugs and side effects. Consequently, we ranked the top 100 high-frequency associations based on the frequency scores predicted by these models. We then compared these rankings with the actual associations in the benchmark dataset to calculate the association prediction accuracy of each method. Meanwhile, SDPred, MGPred, and our method identified the top 100 predicted associations based on association scores.

### Case study

**Fig. 5:**
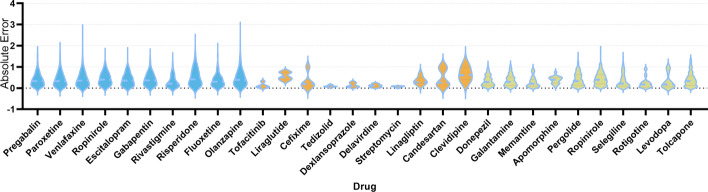
Analysis of variability in predicting drug side effect frequency scores: absolute error. blue represents drugs with the highest incidences of side effects, orange denotes drugs with the lowest incidences, and green indicates a group of drugs for treating Alzheimer’s disease and Parkinson’s disease

Figure 5 uses a violin plot to clearly show the distribution of absolute errors in predicting the frequency scores of side effects for various drugs. We analyzed 30 drugs grouped into three categories: those with the highest and lowest side effect incidences, and those used for treating Alzheimer’s and Parkinson’s diseases. Each violin in the plot represents a specific drug, illustrating the spread and concentration of the absolute errors. The *x*-axis categorizes the drugs, and the *y*-axis measures the absolute errors in predicting each drug’s side effect frequencies. The observed trend suggests that narrower, taller violins correlate with more consistent predictions, whereas wider violins indicate higher variability in accuracy.

**Fig. 6:**
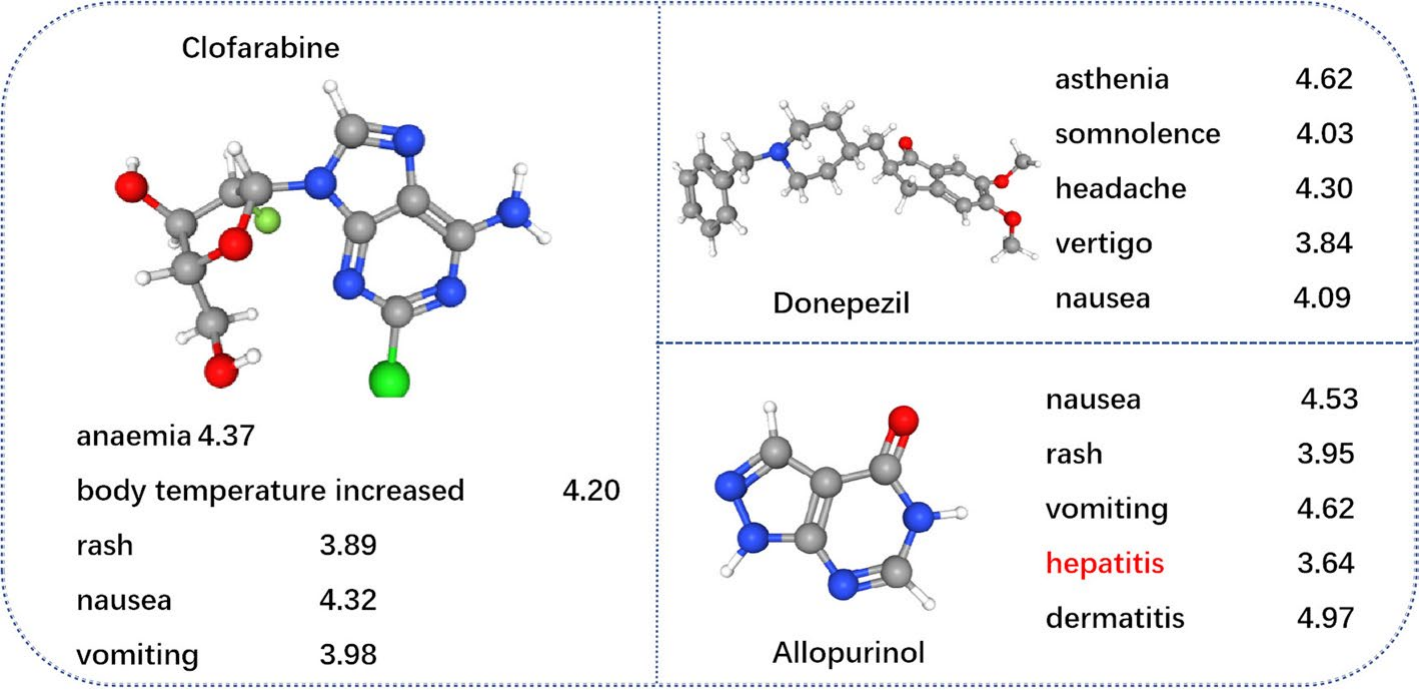
Top *k* predicted side effects of three drugs: Clofarabine, Donepezil and Allopurinol

To examine our model’s ability to predict drug side effect frequencies, we conducted a detailed analysis of three drugs: *allopurinol*, *donepezil*, and *clofarabine*. In our dataset, *allopurinol* has the fewest side effects, while *clofarabine* has the most. Additionally, we specifically investigated the potential side effects of *donepezil* in the context of Alzheimer’s disease. We focused on the five side effects with the highest predicted scores for each drug, as illustrated in Fig. 6. The model proves effective in predicting side effects, even for drugs with minimal side effects, highlighting its robustness. It’s important to mention that in the “ground-truth” dataset, *allopurinol* was not associated with hepatitis. However, our model accurately identified this connection, corroborating the findings of Iqbal et al. [31]. It indicates our model’s ability to successfully detect previously unrecognized potential side effects.

## Conclusion

In this paper, we presented a hybrid multi-modal fusion framework for predicting the frequency of drug-related side effects. We made the first attempt to model the biomedical text of drugs and side effects as new modalities and proposed two multi-modal fusion strategies with different granularities, offering complementary benefits. Our method outperformed existing state-of-the-art models in predicting drug side effect frequency. Ablation experiments confirmed the effectiveness of utilizing multi-modal information, including biomedical text, molecular structure, and attribute similarity, in predicting drug side effects, especially in cold start scenarios. Through case studies and visual analysis, we confirmed the reliability of our hybrid multi-modal fusion framework (HMMF) in predicting side effects of each drug and its ability to detect previously unrecognized potential side effects.

This research has broad applications in drug development, clinical decision-making, public health regulation, and personalized medicine. It accurately predicts drug side effects, offering valuable references to researchers for the discovery and development of safer, more effective drugs, ultimately enhancing treatment outcomes for patients. Simultaneously, this research provides precise medication guidance for clinicians, reducing the incidence of adverse drug reactions and enhancing patient quality of life. In personalized medicine, it contributes to advancing the medical field toward greater precision and personalization, facilitating targeted treatment schemes for individual patients.

While our proposed method has enhanced the performance in identifying the frequency of drug-related side effects, there is still room for improvement. In the future, we plan to explore more effective representation models to uniformly encode the multi-modal information. It’s worth noting that this hybrid multi-modal fusion framework has the potential for extension to other tasks, such as DDI (Drug-drug interaction), DTI (Drug-target interaction), and DTA (Drug-target afnity), by leveraging their rich biological and chemical semantic information.

## Data Availability

The code and data supporting this study and required to reproduce all published results are publicly available on GitHub at https://github.com/catly/HMMF.

## Abbreviations

DDI: Drug-drug interaction
DTA: Drug-target afnity
AUROC: Area under receiver operating characteristic curve
AUPR: Area under the precision-recall curve
RMSE: Root mean squared error
GAT: Graph attention network
SMILES: Simplifed molecular input line entry system
ADR: Adverse drug reaction
CNN: Convolutional neural network
